# DepthLux: Employing Depthwise Separable Convolutions for Low-Light Image Enhancement †

**DOI:** 10.3390/s25051530

**Published:** 2025-03-01

**Authors:** Raul Balmez, Alexandru Brateanu, Ciprian Orhei, Codruta O. Ancuti, Cosmin Ancuti

**Affiliations:** 1 Department of Computer Science, University of Manchester, Manchester M13 9PL, UK; raul.balmez@student.manchester.ac.uk (R.B.); alexandru.brateanu@student.manchester.ac.uk (A.B.); 2 Faculty of Electronics, Telecommunications and Information Technologies, Polytechnic University Timisoara, 300223 Timisoara, Romania; ciprian.orhei@upt.ro

**Keywords:** image sensor restoration, low-light enhancement, vision transformer

## Abstract

Low-light image enhancement is an important task in computer vision, often made challenging by the limitations of image sensors, such as noise, low contrast, and color distortion. These challenges are further exacerbated by the computational demands of processing spatial dependencies under such conditions. We present a novel transformer-based framework that enhances efficiency by utilizing depthwise separable convolutions instead of conventional approaches. Additionally, an original feed-forward network design reduces the computational overhead while maintaining high performance. Experimental results demonstrate that this method achieves competitive results, providing a practical and effective solution for enhancing images captured in low-light environments.

## 1. Introduction

**Figure 1:**
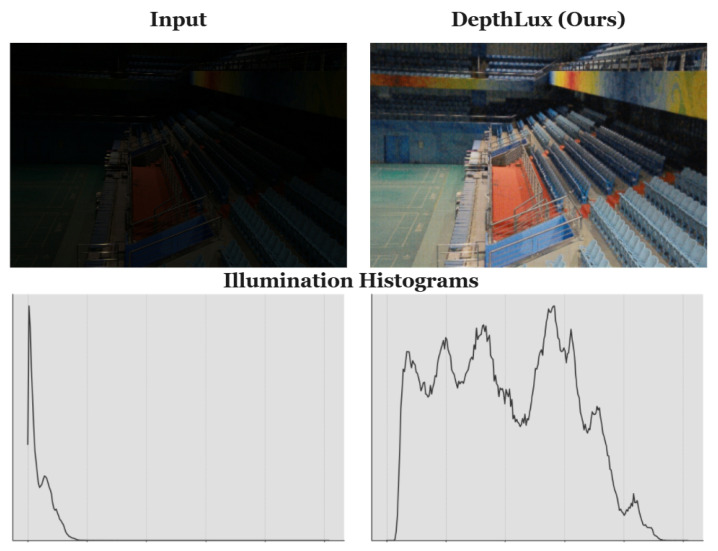
Visual representation of a low-light image and its enhanced counterpart, along with their illumination histograms. We assume illumination to be the maximum value across the channels of each pixel.

Image sensors frequently encounter difficulties in low-light environments, including increased noise and diminished contrast, both of which negatively impact image quality. Low-light image enhancement (LLIE) plays an important role in computer vision (CV), focusing on enhancing the illumination—as shown in Figure 1, contrast, quality, and clarity of images affected by limited visibility and distortions in challenging illuminated settings [1]. Under low-light conditions, image sensors often struggle with noise and diminished contrast, which compromise overall image quality. This degradation not only leads to visually unappealing results but also negatively impacts the functionality of many CV systems.

Low-light environments are characterized by insufficient illumination levels, which often introduce other issues like artifacts and noise. These visual defects are determined by the strong relationship between the colors and the brightness, where the latter can strongly influence the representation of the chromatic element. As the generic low-light environment is not a uniform space in terms of the light distribution, multiple methods have been attempted to demonstrate their ability to enhance images captured under very different conditions [2,3].

Well-established LLIE techniques, such as gamma correction and histogram equalization [1], enhance the illumination factor in images but do not mitigate the other deficiencies introduced by the absence of light. Other classical CV methods attempt to improve these techniques by incorporating illumination factors but often result in unwanted artifacts and imbalances in the enhanced images [1]. A widely adopted approach leverages cognitive models inspired by Retinex theory [4]. The foundational Retinex algorithm is based on selecting an appropriate surround function to determine pixel weighting within the neighborhood of a target pixel, leading to the development of the single-scale Retinex algorithm [5]. This was later expanded upon with multiscale Retinex algorithms [6,7].

The Retinex theory was first mirrored in the deep learning (DL) approaches through the use of CNNs which provided the first well-established LLIE solutions. These methods often struggle to adapt to different light settings, as they rely on a one-to-one mapping in the enhancement process and tend to underfit in the absence of long training times. On the other hand, CNNs successfully manage to capture local contexts but struggle to learn the light patterns represented by long-range dependencies.

The Transformer’s self-attention mechanism [8], later adapted for Vision Transformers (ViTs) [9], offers a partial remedy to these limitations in image processing tasks. However, ViTs face notable drawbacks, including prohibitive computational overhead and resource inefficiency, stemming from the quadratic scaling of self-attention and the intensive use of dense and convolutional layers within feed-forward networks (FFNs).

Depthwise separable convolutions (DSCs) have emerged as an innovation design of CNNs, particularly for applications requiring computational efficiency and reduced model size. This technique decomposes the standard convolution operation into two distinct processes: depthwise convolution and pointwise convolution. The primary advantage of this separation is the significant reduction in the number of parameters and computational complexity, making it particularly suitable for deployment on resource-constrained devices such as mobile phones and embedded systems [10].

The challenge of addressing low-light deficiencies has often been met with computationally intensive solutions, characterized by large parameter counts and intricate mechanisms. We propose a reimagination of the FFN, which traditionally relies on computationally expensive layers to enhance feature representations. Our work demonstrates that DSC can serve as an efficient alternative, maintaining performance integrity while drastically reducing complexity.

Building on the methods and insights presented in [11], we extend this to provide additional motivation for the proposed approach and contextualize the task further. In addition, we introduce a qualitative results section to highlight the perceptual appeal of the enhanced images and conduct an ablation study to justify architectural and training choices. The key innovations introduced by DepthLux can be summarized as follows:

- Reimagining the structure of the FFN, Depthwise Separable Convolutions (DSCs) are employed exclusively as a substitute for traditional convolutional and fully connected layers, significantly enhancing the model’s scalability and computational cost.
- Competitive results are presented while having fewer model parameters than most of the current LLIE methods.
- To address the computational bottleneck of the ViT’s self-attention mechanism, a contraction–expansion strategy is applied, compressing the feature space prior to attention map computation and restoring it afterward, thereby reducing the computational overhead.

The paper is organized as follows: Section 1 introduces the research and highlights its significance. Section 2, titled “Related Work”, provides an overview of the LLIE domain. In Section 3, “Methods”, we describe the proposed model and its key components. Section 4 presents the results obtained on the LOL dataset [12]. Finally, the paper concludes with an ablation study in Section 5 and a discussion of future research directions in Section 6.

## 2. Related Work

LLIE has progressed from traditional CV techniques to advanced DL frameworks, each focusing on different methods to solve the problem. The early methods relied on histogram equalization, gamma correction, and physical models that corrected illumination by estimating environmental light and guided filtering [13]. Retinex-based approaches [4,14,15,16] addressed illumination correction by decomposing images into two components: a high-frequency component (reflectance) and a low-frequency component (illumination). These methods often used mathematical frameworks [17,18], commonly known as Retinex-based variational methods. However, they frequently overlooked noise and artifacts present in low-light environments, limiting their practical effectiveness.

The introduction of DL fundamentally shifted the paradigm in LLIE. CNN-based approaches, exemplified by LLNet [19], pioneered this transformation, followed by advancements such as EnGAN [20], which employed generative models to directly produce normal-light images from low-light inputs. Unsupervised methods, such as Zero-DCE [21], were also introduced, which use differentiable curve estimation and remove the need for paired data but struggle with color fidelity under extreme conditions.

Wei et al. [12] extended Retinex theory to DL, proposing a decomposition framework to enhance illumination and reconstruct well-lit images. Despite their progress, these CNN-driven methods often require complex training procedures and substantial computational resources. Furthermore, their local receptive fields restrict their ability to model global interactions and to capture long-range dependencies, a critical requirement for handling diverse low-light scenarios.

In recent years, Transformers have emerged as a powerful alternative to CNNs, reshaping image restoration tasks [22,23,24,25]. Unlike CNNs, Transformers such as ViTs [9] utilize self-attention mechanisms to capture long-range dependencies, providing a significant advantage in LLIE. Transformer-based models like UFormer [26], Restormer [27], Retinexformer [28], and LytNet [2] have demonstrated this capability effectively while also proving that the effectiveness of the Transformer architecture can also be found in computationally efficient methods. UFormer reimagines the U-Net architecture [29] by substituting convolutions with Transformer blocks while maintaining a hierarchical encoder–decoder structure. KAN-T [30] introduces an innovative attention mechanism based on Kolmogorov–Arnold networks and seamlessly integrates it into a Transformer architecture.

Retinexformer further advances this field by leveraging illumination-based representations to model non-local interactions in regions with varying lighting conditions. The presented methods also offer lightweight solutions, with the total number of trainable parameters ranging from 1.61 million to 26 million. DepthLux, in particular, has 9.75 million parameters, striking a balance between efficiency and performance. This highlights the importance of prioritizing computational efficiency in the design of ViTs for image restoration, ensuring that the models remain practical for real-world applications without compromising quality.

Computational efficiency has also become a focal point in LLIE research. DSCs, for instance, offer an elegant solution to reduce computational complexity by splitting standard convolutions into depthwise and pointwise operations [10,31]. While the depthwise operation applies a filter per channel, the pointwise convolution combines these outputs using 1×1 convolutions, achieving a balance between efficiency and performance. This approach not only reduces the number of parameters but also accelerates inference, making it ideal for real-time applications.

Attention mechanisms have played a pivotal role in advancing LLIE by highlighting features corresponding to poorly lit regions. However, their high computational cost has presented challenges. Transformer-based methods address this issue through innovative strategies such as reducing the dimensions of the attention map to C×C, where *C* is the feature depth [27], or employing feature contraction and expansion techniques around attention computations [2]. These approaches effectively balance performance with efficiency.

The evolution of LLIE methods reflects a continuous effort to bridge the gap between theoretical advances and practical applications. Traditional methods, while foundational, lack robustness in real-world scenarios. CNN-based approaches have marked a significant leap forward but remain constrained by their inability to capture global dependencies. Transformers, with their self-attention mechanisms, have emerged as a transformative force, addressing these limitations and paving the way for more sophisticated LLIE frameworks.

## 3. Methods

**Figure 2:**
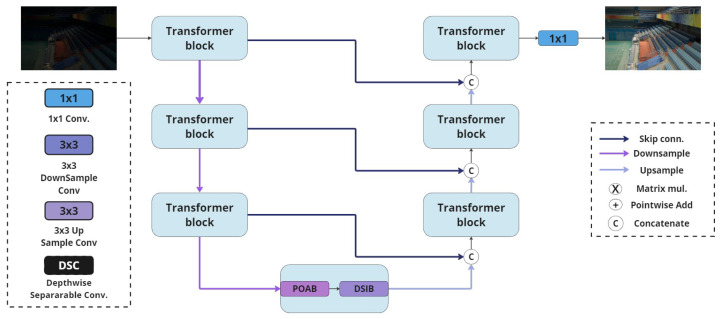
The architecture of DepthLux introducing a conventional Transformer architecture enhanced with the POAB and the DSIB.

Our approach demonstrates that standard convolutions, commonly employed in most LLIE ViTs, can be efficiently replaced with depthwise separable convolutions (DSCs), yielding minimal performance degradation while significantly reducing model parameters. Based on a conventional transformer framework as illustrated in Figure 2, DepthLux represents a paradigm shift in the traditional design of feed-forward networks (FFNs) by introducing the Depthwise Separable Illumination Block (DSIB). Additionally, it aims to optimize the self-attention mechanism through the Performance-Optimized Attention Block (POAB).

### 3.1. Depthwise Separable Illumination Block

The innovation introduced in the Depthwise Separable Illumination Block (DSIB), which forms the core of DepthLux’s FFNs, lies in its exclusive reliance on DSCs. This design choice plays a pivotal role in achieving the computational efficiency of the model. To illustrate, consider a convolution operation with a kernel of size $D_{k}\times D_{k}$, *M* input channels, and *N* output channels. A standard convolution requires $D_{k}\times D_{k}\times M\times N$ multiplications, as each output channel computes a weighted sum over the entire input space. In contrast, a depthwise separable convolution significantly reduces this complexity by factorizing the operation into two distinct steps: (1) a depthwise convolution, which applies a single convolutional filter to each input channel independently, and (2) a pointwise convolution, which combines these filtered outputs across channels. As a result, the total number of multiplications required by a DSC is reduced to $D_{k}\times D_{k}\times M+M\times N$, a substantial improvement over standard convolutions. Letting $x_{m}$ represent the input channels, *k* the convolution kernel, $k^{\mathrm{depthwise}}$ and $k^{\mathrm{pointwise}}$ the depthwise and pointwise convolution kernels, and $y_{n}$ the output, we can compare the functionality of the convolution and the DSC as follows:

(1) $$\;\mathrm{Convolution}:\;y_{n}=\sum_{m=1}^{M}\left(x_{m}*k\right),\;\forall n\in \left\{1,\cdots ,N\right\},$$

(2) $$\mathrm{DS}\;\mathrm{Convolution}:\;y_{n}=\sum_{m=1}^{M}\left(\left(x_{m}*k^{\mathrm{depthwise}}\right)*k^{\mathrm{pointwise}}\right),\;\forall n\in \left\{1,\cdots ,N\right\},$$

**Figure 3:**
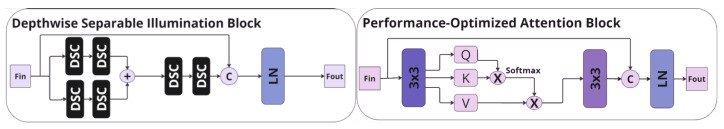
DSIB, which represents the feed-forward section of our proposed Transformer, exclusively uses DSC (**left**). POAB is built to reduce the computational complexity of the model by contracting and then expanding the feature space (**right**).

The integration of the 3×3 kernel, $k^{\mathrm{depthwise}}\in R^{3\times 3}$ for depthwise convolutions (DWConv), alongside the 1×1 kernel, $k^{\mathrm{pointwise}}\in R^{1\times 1}$, for pointwise convolutions (PWConv), enables a comprehensive extraction of features across varying spatial scales while maintaining computational efficiency. The parallel configuration of depthwise separable convolutions (DSCs) at the onset of the DSIB (illustrated in Figure 3) is explicitly designed to model diverse, multiscale illumination patterns. This architecture enables the extraction of rich illumination features across various scales, which are subsequently aggregated through the summation of the feature maps:

(3) $$F_{\mathrm{in}}^{\prime }=\sum_{i=1}^{2}{\mathrm{DSC}}^{\left(i\right)}\left({\mathrm{DSC}}^{\left(i\right)}\left(F_{\mathrm{in}}\right)\right),\;F_{\mathrm{in}}^{\prime }\in R^{H\times W\times C}.\;$$

The fused map is further processed and refined through another set of DSC, which extracts the most relevant information. To transmit information to future layers, we employ a skip connection originating from $F_{\mathrm{in}}\in R^{H\times W\times C}$, which is concatenated (Equation (4)) with the output processed using multiple DSCs:

(4) $$F_{\mathrm{out}}=\left[F_{\mathrm{in}},\mathrm{DSC}\left(\mathrm{DSC}\left(F_{in}^{\prime }\right)\right)\right],\;F_{\mathrm{out}}\in R^{H\times W\times 2C}\;$$

DepthLux introduces the innovative application of the Gaussian Error Linear Unit (GELU) activation following each stage of the DSC. By modulating inputs according to their magnitude and alignment with the Gaussian distribution mean, GELU ensures the preservation of nuanced features even in noise-contaminated settings, offering a distinct advantage over traditional activation functions:

(5) $$\mathrm{DSC}\left(F_{\mathrm{in}}\right)=\mathrm{GELU}\left(\mathrm{PWConv}\left(\mathrm{GELU}\left(\mathrm{DWConv}(F_{\mathrm{in}})\right)\right)\right)\;$$

### 3.2. Performance-Optimized Attention Block

Figure 3 provides a schematic representation of the POAB block. The proposed methodology aims to establish that models with a significantly reduced computational overhead can achieve performance levels comparable to state-of-the-art approaches. This concept is exemplified within the attention module.

The input feature tensor $F_{\mathrm{in}}\in R^{H\times W\times C}$ is first reduced in resolution by a factor of four through a 3×3 convolution, producing a condensed representation:

$$F_{\mathrm{down}}=\mathrm{conv}{3\times 3}_{\mathrm{down}}\left(F_{\mathrm{in}}\right),\;F_{\mathrm{down}}\in R^{H/4\times W/4\times C/2}$$

Following this compression, the fundamental self-attention components—query (Q), key (K), and value (V)—are computed via fully connected layers as described in Equation (6):

(6) $$\begin{array}{cc} Q & =W_{Q}\cdot F_{\mathrm{down}};\;K=W_{K}\cdot F_{\mathrm{down}};\;V=W_{V}\cdot F_{\mathrm{down}}\; \end{array}$$

The attention mechanism responsible for capturing long-range dependencies can be formally expressed as follows, where dim represents the number of output channels used in the computation process of Q,K, and *V*:

(7) $$Attn=\mathrm{Softmax}\left(\frac{QK^{⊤}}{\sqrt{dim}}\right)V,\;Attn\in R^{H/4\times W/4\times C}\;$$

Upon calculating the attention map, the feature space is restored to its original dimensions using a 3×3 transposed convolution:

(8) $$\begin{array}{c} Attn_{\mathrm{out}}=\mathrm{conv}{3\times 3}_{\mathrm{up}}\left(Attn\right),\;Attn_{\mathrm{out}}\in R^{H\times W\times C} \end{array}$$

To preserve spatial information during the attention map computation, the input feature tensor $F_{\mathrm{in}}\in R^{H\times W\times C}$ is routed through a skip connection, ensuring its integration into deeper layers. This tensor is subsequently concatenated with the attention output $Attn_{\mathrm{out}}\in R^{H\times W\times C}$ which is expanded as formalized in Equation (8). The resulting feature map undergoes layer normalization, which aligns the feature distributions to ensure stability and consistency across channels.

The expansion–contraction strategy helps reduce the computational cost of the attention mechanism. By downsampling $F_{\mathrm{in}}\in R^{H\times W\times C}$ to $F_{\mathrm{down}}\in R^{H/4\times W/4\times C/2}$, we also reduce the depth of the features, optimizing the attention mechanism. While this leads to some information loss, fully mitigating it would require quadrupling the number of output channels to maintain the number of elements between the input feature map and the downsampled map. However, this would result in an additional 42 million parameters, which would significantly increase the model’s complexity.

## 4. Results

### 4.1. Implementation Details

The architecture adopts a standard transformer-based design, comprising three encoder–decoder pairs and a bottleneck block that serves as both a refinement module and a bridge between the encoding and decoding phases. The dimensional settings for the encoders and their respective decoders are specified as [64,128,256], where each value represents the embedding size, which determines the number of features in each layer. The bottleneck block operates with a fixed embedding size of 256, maintaining consistency in feature representation at the network’s deepest level. In addition, the transformer follows a U-Net structure: after each encoder, a downsampling operation reduces the spatial resolution to capture higher-level features, and before each decoder, an upsampling step is applied to restore the resolution. This arrangement ensures that the network effectively integrates the global context with local detail during both the encoding and decoding processes.

### 4.2. Training Details

DepthLux is tailored to handle a diverse range of images affected by insufficient illumination. To rigorously evaluate its performance, experiments are conducted on three benchmark datasets: LOL-v1, LOL-v2 real [32], and LOL-v2 synthetic [32]. Performance is measured using the Peak Signal-to-Noise Ratio (PSNR) and the Structure Similarity Index Measure (SSIM) [33], as shown in Table 1. These datasets represent the current standard for assessing state-of-the-art methods in LLIE. The framework used for the implementation is TensorFlow, and the training is performed on a Nvidia 3090 GPU using image patches sized 256×256 pixels. Data augmentation strategies, such as random cropping and flipping, are employed to enrich the training dataset and enhance model generalization.

The optimization process leverages the Adam optimizer, configured with $\beta_{1}=0.9$ and $\beta_{2}=0.999$. To further guide the training towards superior image restoration quality, a loss function based on PSNR as described in [2] is adopted. This loss function emphasizes the reconstruction of high-fidelity images and is detailed in Equation (9):

(9) $$L_{\mathrm{PSNR}}=40-PSNR\left(\mathrm{predicted},\mathrm{ground}\;\mathrm{truth}\right)$$

To address the challenges posed by local minima during the training of deep neural networks, a learning rate decay strategy is implemented. In particular, a cosine annealing schedule is employed, gradually reducing the learning rate from $2\times 10^{-4}$ to $1\times 10^{-6}$ over the course of training. This scheduling mechanism is designed to promote more stable convergence and improve the overall performance of the model.

### 4.3. Results Discussion

From a quantitative point of view, DepthLux achieves an competitive result which ranks the method second and third on the three tested datasets in terms of PSNR. We achieve an improvement of 9.7 dB against Uformer [26], 3.63 dB against Restormer [27], and 8.58 dB against EnGAN [20], while being 71%, 74%, and 62% more efficient than LLFormer [44], LLFlow [45], and Restormer [27], respectively, in terms of parameter count. However, some limitations in both performance and computational efficiency remain. These issues are highlighted by the gap with LL-SKF [46] across all three tested datasets, as well as the minimal 0.04 dB difference between DepthLux and both LLFormer [44] and LLFlow [45] on the LOL-v2 real datasets.

**Figure 4:**
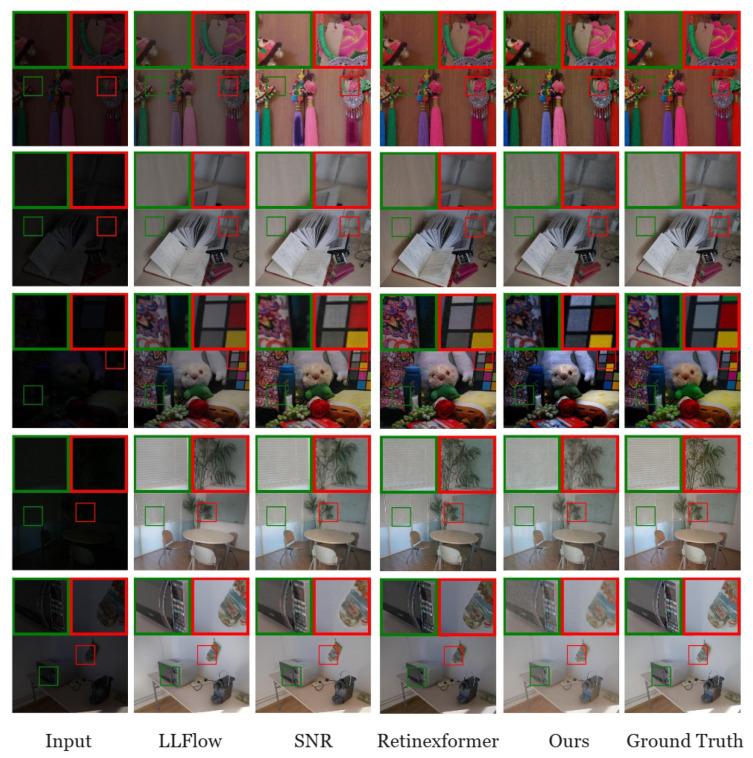
Qualitative results on the LOL dataset [12] comparing DepthLux with other SOTA LLIE methods.

The qualitative results in Figure 4 demonstrate that DepthLux effectively enhances images without introducing artifacts or color distortions, unlike SNR and LLFlow. This highlights its ability to preserve natural image quality while improving visibility. However, due to the design trade-offs made in the POAB block to enhance computational efficiency, a slight graininess can be observed in the enhanced images. While this effect is minimal, it represents a compromise between performance and efficiency.

### 4.4. Scaling Considerations

The results presented confirm that DepthLux achieves parameter efficiency by replacing conventional convolutions with DSC. In the current architecture, employing standard convolutions within the FFN yields a total of 26,402,980 trainable parameters. Substituting these with DSC reduces this count drastically to 9,754,780. To further support this assertion, we evaluate DepthLux’s scalability, examining how varying model sizes impact parameter count when using DSC versus traditional convolutions. This is accomplished by modulating a scaling factor that governs layer dimensions throughout the network.

The parameter growth trend for models utilizing standard convolutions follows a significantly steeper gradient compared to those employing DSC as depicted by their respective curves. This pronounced difference underscores the enhanced scalability of DepthLux.

## 5. Ablation Study

We conduct an ablation study on the LOL-v1 dataset to demonstrate the effectiveness of our proposed framework, and utilize PSNR to measure performance. The ablation study focuses on activation function and loss function variants. The results of the study can be found in Table 2.

To provide further justification for our selection of the loss function in training this model, we conduct a series of experiments comparing the performance of DepthLux when trained using different loss functions. Specifically, we evaluate the model’s performance with three loss functions: $L_{1}$ loss, $L_{2}$ loss, and our proposed $L_{\mathrm{PSNR}}$ loss function.

$L_{1}$ loss minimizes the absolute differences between the predicted and ground truth values, promoting robustness to outliers. $L_{2}$ loss focuses on minimizing squared differences, penalizing larger errors more heavily. The $L_{\mathrm{SSIM}}$ loss, defined as

(10) $$L_{\mathrm{SSIM}}=1-\mathrm{SSIM}\left(y_{\mathrm{target}},y_{\mathrm{predicted}}\right)$$

s focuses on preserving the structural similarity between the enhanced and ground truth images, improving the perceptual quality and structural fidelity. In contrast, our $L_{\mathrm{PSNR}}$ loss directly optimizes the PSNR by aligning the loss function with the evaluation criterion. The $L_{\mathrm{PSNR}}$ loss prioritizes features critical for high-quality reconstructions, leading to superior performance (0.35 dB compared to $L_{1}$, 0.2 dB compared to $L_{\mathrm{SSIM}}$, and 0.05 dB compared to $L_{2}$).

GELU [47] outperforms no activation (by 0.37 dB), ReLU [48] (by 0.08 dB), and PReLU [49] (by 0.06 dB) when used in DSC due to its smooth and probabilistic formulation, which enables better feature discrimination and gradient flow. Unlike ReLU and PReLU, GELU transitions inputs non-linearly while avoiding abrupt changes or gradient instability. This smoothness allows DSC to model complex relationships more effectively, improving feature extraction and stability during training.

## 6. Conclusions and Future Work

**Figure 5:**
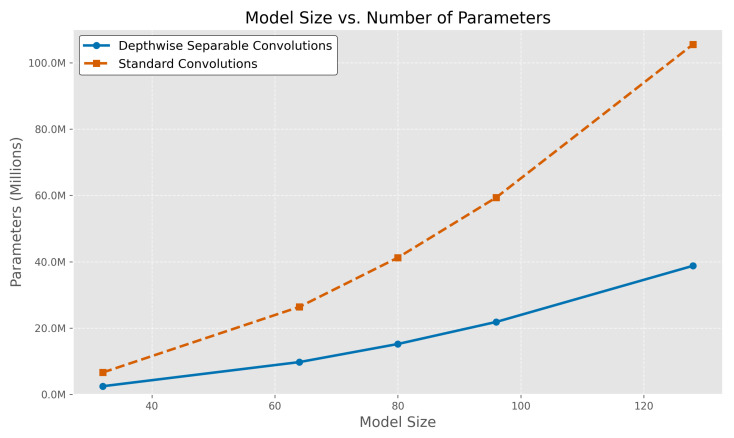
A comparison of the number of parameters and model sizes between models employing DSCs and those utilizing standard convolutions. The model size values correspond to the parameter that regulates the layer dimensions.

This study establishes that ViTs can effectively utilize DSC as a viable alternative to traditional convolutional layers. By integrating DSC into the model architecture, the parameter count is significantly reduced, and computational efficiency is improved, leading to a more resource-conscious design without compromising performance in LLIE tasks. Our analysis from Figure 5 shows that DSC achieves an approximate 2.5× reduction in parameter count compared to standard convolutions, requiring only ~40% of the parameters needed for similar architectures. The reduced complexity introduced by DSC highlights its potential as a cornerstone for developing lightweight and scalable ViT architectures, paving the way for more efficient deployment in real-world applications.

The use of DSC not only reduces computational complexity but also demonstrates positive results on the LOL-v1 and LOL-v2 datasets as highlighted in the manuscript. To further expand our evaluation, we plan to include non-uniformly illuminated image datasets, such as the one provided in [50], in our future work.

Future directions will aim to overcome the current framework’s limitations, focusing on optimizing the trade-off between model size and performance, particularly under extremely resource-constrained conditions. Research efforts will also explore the integration of complementary advancements, such as hybrid attention mechanisms that combine the strengths of different attention paradigms or adaptive depthwise operations tailored to varying input complexities. These innovations hold promise for further enhancing the capability and versatility of ViTs, ensuring they remain at the forefront of LLIE solutions and other computationally demanding tasks.

## Figures and Tables

**Table 1:** Results on LOL-v1 and LOL-v2 datasets and parameter counts of different models. **Red**, **blue**, and **green** metrics represent first, second, and third places, respectively.

Methods	Param (M)	LOL-v1		LOL-v2-R		LOL-v2-S	
		PSNR	SSIM	PSNR	SSIM	PSNR	SSIM
SID [34]	7.76	14.35	0.436	13.24	0.442	15.04	0.610
DeepUPE [35]	1.02	14.38	0.446	13.27	0.452	15.08	0.623
RF [36]	21.54	15.23	0.452	14.05	0.458	15.97	0.632
DeepLPF [37]	1.77	15.28	0.473	14.10	0.480	16.02	0.587
UFormer [26]	5.29	16.36	0.771	18.82	0.771	19.66	0.871
RetinexNet [12]	0.62	16.77	0.462	18.37	0.723	17.13	0.798
IPT [23]	115.31	16.27	0.504	19.80	0.813	18.30	0.811
Sparse [32]	2.33	17.20	0.640	20.06	0.816	22.05	0.905
EnGAN [20]	8.64	17.48	0.652	18.64	0.677	16.57	0.734
RUAS [38]	0.003	18.23	0.720	18.37	0.723	16.55	0.652
FIDE [39]	8.62	18.27	0.665	16.85	0.678	15.20	0.612
DRBN [40]	2.21	19.86	0.834	20.13	0.830	23.22	0.927
KinD [41]	8.03	20.87	0.799	17.54	0.669	16.26	0.591
Restormer [27]	26.13	22.43	0.823	19.94	0.827	21.41	0.830
MIRNet [42]	5.90	24.14	0.842	20.36	0.782	21.94	0.846
SNR-Net [43]	4.01	24.61	0.842	21.48	0.849	24.14	0.928
LLFlow [44]	37.68	25.13	0.872	26.20	0.888	24.81	0.919
Retinexformer [28]	1.61	25.16	0.845	22.80	0.840	25.67	0.930
LLFormer [45]	24.55	25.76	0.823	26.20	0.819	28.01	0.927
LL-SKF [46]	39.91	26.80	0.879	28.45	0.905	29.11	0.953
**DepthLux** (Ours)	9.75	26.06	0.793	26.16	0.794	28.69	0.920

**Table 2:** Ablation experiments on activation functions and loss functions. **Red** metrics represent the best results.

(a) The Effect of Different Activation Functions Used in the DSC.		(b) Impact of Different Loss Functions on Performance.	
Activation	PSNR	Loss Function	PSNR
No Activation	25.70	$L_{1}$	25.71
ReLU	25.98	SSIM	25.86
PReLU	26.00	$L_{2}$	26.01
GELU	**26.06**	$L_{\mathrm{PSNR}}$	**26.06**

## Data Availability

Data are contained within the article.

## Abbreviations

The following abbreviations are used in this manuscript:

CV	Computer Vision
DW-Conv	Depthwise Convolutions
CNN	Convolutional Neural Network

DL	Deep Learning
DSC	Depthwise Separable Convolutions
DSIB	Depthwise Separable Illumination Block
FFN	Feed-Forward Network
GELU	Gaussian Error Linear Unit
K	Self Attention Key
LLIE	Low-light Image Enhancement
LN	Layer Normalization
POAB	Performance-Optimized Attention Block
PReLU	Parametric Rectified Linear Unit
PSNR	Peak Signal-to-Noise Ratio
PW-Conv	Depthwise Convolutions
ReLU	Rectified Linear Unit
Q	Self-Attention Query
SOTA	State of the Art
SSIM	Structural Similarity Index Measure
V	Self Attention Value
ViT	Vision Transformers
